# The Electrical Stimulation of the Bed Nucleus of the Stria Terminalis Causes Oxidative Stress in Skeletal Muscle of Rats

**DOI:** 10.1155/2018/4671213

**Published:** 2018-05-31

**Authors:** Mateusz Jakub Karnia, Dorota Myslinska, Katarzyna Patrycja Dzik, Damian Jozef Flis, Ziemowit Maciej Ciepielewski, Magdalena Podlacha, Jan Jacek Kaczor

**Affiliations:** ^1^Department of Neurobiology of Muscle, Faculty of Rehabilitation and Kinesiology, Gdansk University of Physical Education and Sport, Kazimierza Gorskiego 1, 80-336 Gdansk, Poland; ^2^Faculty of Physical Education, Gdansk University of Physical Education and Sport, Kazimierza Gorskiego 1, 80-336 Gdansk, Poland; ^3^Department of Animal and Human Physiology, Faculty of Biology, University of Gdansk, 80-308 Gdansk, Poland; ^4^Department of Bioenergetics and Nutrition, Faculty of Rehabilitation and Kinesiology, Gdansk University of Physical Education and Sport, Kazimierza Gorskiego 1, 80-336 Gdansk, Poland

## Abstract

Recent studies indicate that activation of hypothalamus-pituitary-adrenocortical axis (HPA) plays the crucial role in stress response, while several lines of evidence mark the bed nucleus of the stria terminalis (BST) as a major mediator of the HPA axis responses to stress. The purpose of this study was to investigate the influence of the corticosterone flux induced by the electrical stimulation of BST on markers of free radical damage of lipids and proteins and antioxidant enzyme activity in skeletal muscle of rats. The male Wistar rats were used and assigned to one of three groups: sham-operated (SHM; *n* = 6), two-week (ST2; *n* = 6), and four-week stimulated (ST4; *n* = 5) groups. Blood, soleus, and extensor digitorum longus muscles were collected. The chronic, 4-week electrical stimulation of the BST evokes increased plasma corticosterone concentration, which resulted in oxidative stress in skeletal muscles. We found higher level of lipid peroxidation markers, lower level of protein oxidation marker, and elevated antioxidant enzyme activity in both muscles. Our findings have also potential implication showing that reaction to the long-term “psychological stress” may lead to free radical damage of muscle.

## 1. Introduction

It is well known that chronic stress response has extremely negative consequences for health. However, the molecular mechanisms that trigger and accelerate the process of stress inducing life-threatening effects, death, and the pathophysiological complications of diseases that arise from stress has not been definitively clarified. A number of studies have focused on the understanding of molecular pathways that determine system response to stress through the release of glucocorticoids (GCs) [1, 2]. The catabolic effects of GCs are well known, and destructive role of GCs is manifested in skeletal muscle disorders, including oxidative stress and muscle atrophy [3–8].

The oxidative stress is defined as a disturbance of prooxidant-antioxidant balance in the direction of oxidation reaction. This stress is a series of biochemical reactions that result in free radical cell damage or its compartments by reactive oxygen and nitrogen species (RONS). Mitochondria and cytoplasm are the main sources of reactive oxygen species (ROS) generation in the skeletal muscle [3]. Recently, it has been shown that both sources of ROS generation are involved in aging process and dysfunction in several metabolic and neurodegenerative diseases, which may be partially connected with elevated GC level [9]. A growing body of evidence demonstrates that GC signaling is a common mediator of wasting, irrespective of the underlying initiator or disease state. The GCs may increase a leakage of protons from mitochondria, thus affecting the electron transport chain activity, mitochondrial membrane potential, and ATP generation, and may lead to cell injury and cell death [4]. Furthermore, high plasma levels of GCs can alter the physicochemical properties of a biological membrane, particularly cellular and mitochondrial membranes. Recently, it has been presented that GCs may penetrate into the membrane and change functions of membrane proteins, thereby affecting lipid peroxidation and membrane permeability [5], protein carbonylation, and mitochondrial dysfunction [6, 7]. Many previous data have reported the inductive role of GCs on the oxidative stress in the variety of different tissues such as the brain [10–13], liver [11–14], heart [11], bone [9], tendons [15], and muscles [8, 16]. Moreover, disturbances in oxygen metabolism and downregulation of cell's antioxidant capacity are considered to be an important factor in the development of neurodegenerative disorders such as Alzheimer disease (AD) and Parkinson disease (PD) and amyotrophic lateral sclerosis (ALS) [17]. In addition, Sato and coworkers have indicated the direct link between the increased level of GCs with ROS generation and the progression of the neurodegenerative diseases [10].

It is generally known that one of the main mechanisms responsible for the release of GCs is hypothalamus-pituitary-adrenocortical axis (HPA) activation [18]. HPA is a critical component of the body's stress response. There is an evidence indicating the bed nucleus of the stria terminalis (BST) to be a major mediator of the HPA axis in the response to stress [19, 20]. Moreover, some data suggest that the BST acts as a relay between limbic processing of emotional information with a final response of the HPA axis [21]. The possible connections act mainly through sending projections from the BST to the corticotrophin-releasing hormone regions of the paraventricular hypothalamic nucleus [22].

Despite the fact that oxidative stress in skeletal muscles has been shown to be induced by GCs, there are no reports regarding the influence of chronic stress response to endogenous GC action on oxidative stress in skeletal muscles through the direct HPA axis activation. Additionally, some previous reports suggest that treatment with exogenous GCs to simulate a stress response may not reflect real state of stress response. The purpose of this study was to determine the chronically persistent endogenous GC release (chronic stress response) induced by the electrical stimulation of the BST on markers of free radical damage of lipids and protein and antioxidant enzyme activity in two different types of the skeletal muscles in rat. We believe that advantage of our model is that it mimics unconscious stress state in stimulated rats [23, 24]. We hypothesize that chronic stress response (CSR) linking with GC action may influence on the oxidative damage in the skeletal muscle in rat.

## 2. Materials and Methods

### 2.1. Animals

Male *Wistar* rats (250–300 g) were used. The animals were provided a standard diet *ad libitum* with free access to water and were maintained on a 12 h light/dark cycle, temperature 22°C, and humidity 50–55%. The animals were habituated daily for about two weeks before the experiment to minimize stress caused by the experimental procedures. In brief, the habituation were carried out in a sound attenuating chamber in a 250 × 350 × 440 mm testing box. The rats were taken from their home cages and placed in the testing box where they had free access to food and were allowed to explore the box for 30 min. Experimental procedures were performed between 8 : 00 and 13 : 00. The rats were divided randomly into three groups: the BST two-week electrically stimulated group (ST2; *n* = 6), the BST four-week electrically stimulated group (ST4; *n* = 5), and the BST sham group (SHM; operated but not stimulated; *n* = 6).

The experiments were carried out in accordance with the European Communities Council Directive (86/609/EEC), and the protocols were approved by the Local Animal Research Ethical Committee for the Care and Use of Laboratory Animals at the Medical University in Gdansk, Poland (number 8/2010).

### 2.2. Surgery

Standard stereotaxic surgery was performed under pentobarbital anesthesia (60 mg/kg i.p.) (Vetbutal, Biowet Puławy, Poland) with a premedication of xylazine (5 mg/kg i.p.) (Sedazin, Biowet, Puławy) and 0.1% solution of atropine (0.25 mg per animal) according to Myślińska and coworkers [25].

### 2.3. The Electrical Stimulation

The electrical stimulation was performed following the procedure described before [25]. Briefly, two weeks after recovery from the surgery, the stimulated groups were screened for BST stimulation-induced behavior. Once determined, the current intensity was held constant throughout 14 (ST2) or 28 (ST4) consecutive days of the stimulation. The animals from the SHM group were treated in the same way as the experimental group with the exception that no current was passed through the electrodes.

### 2.4. Animal Sacrifice, Blood, and Muscle Collection

All animals were sacrificed at required time point (one hour after termination of the last electric stimulation). The blood samples were collected by heart puncture one hour after the last session of stimulation. The blood was centrifuged at 2000*g* for 10 min at 4°C. Plasma was separated and stored at −80°C for later analysis. Extensor digitorum longus (EDL) and soleus (SOL) muscles were removed from both hind limbs, dissected from fat and connective tissue, and placed into individual tubes for immediate freezing in liquid nitrogen. All samples were stored at −80°C until analysis. After sacrificing, rats' brains had perfused by 4% polyformaldehyde and removed in order to carry out histological verification of the electrodes' placement.

### 2.5. Muscle Homogenization

Prior to the chemical assays, muscles were minced and homogenized in an ice-cold buffer that contained 50 mM potassium phosphate, 1 mM EDTA, 0.5 mM DTT, 1.15% KCl, and 1 : 200 protease inhibitor (Sigma-Aldrich, P8340) at pH 7.4. The homogenates were then centrifuged at 750*g* at 4°C for 10 min, then supernatants were collected and stored in −80°C until analysis. Protein concentration was determined using the Bradford assay (Sigma-Aldrich, B6916) according to manufacturer's instructions.

### 2.6. Plasma Corticosterone (CORT)

The plasma CORT concentration was determined by radioimmunoassay using a commercially available kit (Rat corticosterone 125I RIA kit, Institute of Isotopes, Budapest, Hungary) and Wizard 1470 gamma counter. The concentration of CORT was expressed as nanogram per ml of plasma.

### 2.7. Enzyme Activities and Oxidative Stress Markers in Skeletal Muscles

#### 2.7.1. Superoxide Dismutase (SOD)

The muscle SOD activity was determined in the SOL and EDL muscles by measuring the kinetic consumption of O_2_^−•^ by superoxide dismutase in a competitive reaction with cytochrome c, as described by Flohé and Ötting [26]. In the SOL muscle, 7.5 *μ*l of supernatant was added to a cuvette containing 982.5 *μ*l of medium (50 mM phosphate buffer, 1 mM EDTA, pH 7.8, with partially acetylated cytochrome c (25 mg/100 ml)) and xanthine (0.5 *μ*M). In the EDL muscle, 15 *μ*l of supernatant and 975 *μ*l of medium were added. 10 *μ*l of xanthine oxidase (0.2 U/ml) was added to initiate the reaction, and absorption was measured at 550 nm for 3 min at 30°C. In a separate cuvette, MnSOD activity was measured on the same sample analyzed under identical conditions with the addition of 10 *μ*l of 200 mM KCN (prepared fresh daily at pH 8.5–9.5). Cu/ZnSOD activity was calculated by subtracting MnSOD activity from total SOD activity. The SOD activities were expressed as units per milligram of protein.

#### 2.7.2. Catalase (CAT)

The muscle CAT activity was measured in the SOL and EDL muscles by estimating the kinetic decomposition of H_2_O_2_, according to Aebi [27]. Briefly, 30 *μ*l of supernatant of the SOL or 100 *μ*l of the EDL from the 750*g* spin was added to a cuvette containing 960 *μ*l or 890 *μ*l of medium, respectively (50 mM phosphate buffer, 5 mM EDTA, and 0.05% Triton X-100 at pH 7.4). 10 *μ*l of 1 M H_2_O_2_ was added to the cuvette and mixed to initiate the reaction. Absorbance was measured at 240 nm for 1 min at 30°C. CAT activity was expressed as micromoles per minute per milligram of protein.

All of the samples were analyzed in duplicate, and all kinetics were measured in a temperature-controlled Cecil Super Aquarius CE 9200 spectrophotometer.

#### 2.7.3. Glutathione Peroxidase (GPx)

The GPx activity in muscle homogenates was determined by Glutathione Peroxidase Kit (703102, Cayman Chemicals, USA) in accordance with supplied manufacturer's instruction.

The activity of GPx was expressed as nanomoles per minute per milligram of protein.

#### 2.7.4. 8-Isoprostanes (8-iso)

A marker of lipid peroxidation, skeletal muscle 8-iso content, was determined with 8-Isoprostane ELISA Kit (516351, Cayman Chemicals, USA) according to the manufacturer's instruction. The concentration of 8-iso was expressed as picograms per milligram of protein.

#### 2.7.5. Glutathione Disulfide (GSSG)

GSSG was determined by using a kinetic assay as described by Akerboom and Sies [28]. In brief, the homogenate was centrifuged at 5000*g* for 5 min, the supernatant was neutralized in 300 mM 3-(N-morpholino) propanesulfonic acid in 2 M solution of KOH. The samples were analyzed in the medium containing 100 mM potassium phosphate buffer pH 7.0, 1 mM EDTA, 0.1 mM 5.5′-dithio-bis (2-nitrobenzoic acid) (DTNB), 0.2 U/ml glutathione reductase, and 0.2 mM NADPH and were measured by using the spectrophotometer (Cecil Super Aquarius CE 9200) at 412 nm. A standard curve made of fresh GSSG was used to calculate the concentrations of GSSG. The concentration of GSSG was expressed as nanomoles per milligram of protein.

#### 2.7.6. Sulfhydryl Group Content (SH Groups)

The SH group content in the muscle homogenates was measured spectrophotometrically (Cecil Super Aquarius CE 9200) with DTNB assay according to a previously described procedure [29]. Briefly, samples were incubated with 0.1 mM DTNB at room temperature for 60 min. Absorbance was determined at 412 nm. The level of SH groups was expressed as micromoles per gram of tissue.

#### 2.7.7. Malondialdehyde (MDA)

The muscle MDA level was determined as previously described [30]. In brief, the absorbance of the 750*g* centrifuged homogenate was measured on the spectrophotometer (Cecil Super Aquarius CE 9200) at 586 nm. The level of MDA in the samples was determined using 10 mM 1,1,3,3-tetramethoxypropane as a standard. The level of MDA was expressed as micromoles per gram of tissue.

#### 2.7.8. Statistical Analysis

Statistical analysis was performed using a software package (Statistica v. 12.0, StatSoft Inc., Tulsa, OK, USA). The results are expressed as mean ± SD. The differences between groups were tested using one-way ANOVA followed by Tukey post hoc test; *p* values less than 0.05 were considered statistically significant.

## 3. Results

### 3.1. Serum CORT Level

**Figure 1:**
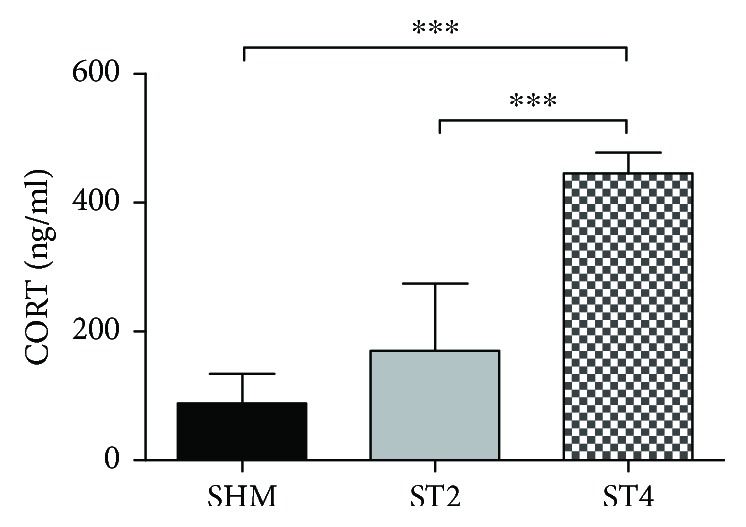
The level of CORT in plasma. Results were expressed as mean ± SD, SHM (*n* = 6), ST2 (*n* = 6), ST4 (*n* = 5). ^∗∗∗^*p* < 0.001.

Serum CORT concentration after BST stimulation was significantly higher in the ST4 group as compared with the ST2 and SHM groups (*p* < 0.001; Figure 1). The CORT concentration was 445.9 ± 31.6, 170.1 ± 104.1, and 88.6 ± 45.6 ng/ml in the ST4, ST2, and SHM groups, respectively.

### 3.2. Oxidative Stress Markers and Enzyme Activities in Skeletal Muscles

#### 3.2.1. Markers of Lipid Peroxidation

**Figure 2:**
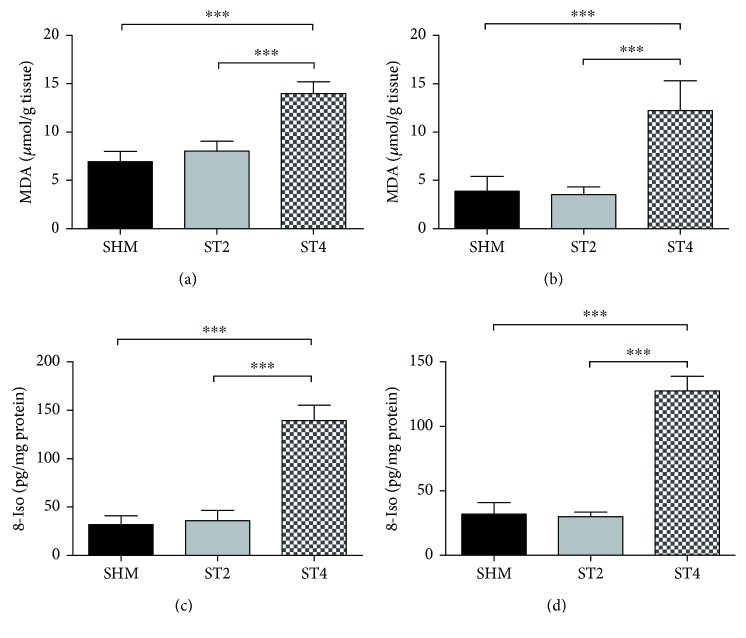
The level of MDA in (a) SOL and (b) EDL. The level of 8-iso in (c) SOL and (d) EDL. Results were expressed as mean ± SD, SHM (*n* = 6), ST2 (*n* = 6), ST4 (*n* = 5). ^∗∗∗^*p* < 0.001.

The level of both lipid peroxidation markers (MDA and 8-iso) in the SOL and EDL muscles were significantly elevated in the ST4 group compared with the other groups. The concentration of MDA was significantly higher in the ST4 (14.0 *μ*mol/g tissue) than ST2 and SHM groups in the SOL muscle (8.0, *p* < 0.001 and 6.9 *μ*mol/g tissue, *p* < 0.001, resp.) (Figure 2(a)). After 4 weeks of the BST electrical stimulation, the concentration of MDA was also elevated in the ST4 group as compared to both ST2 and SHM groups in the EDL muscle (3.5-fold, *p* < 0.001 and 3-fold, *p* < 0.001, resp.); (Figure 2(b)). The level of 8-iso in the SOL muscle was the highest in the ST4 group (139.4 ± 16.0) as compared with the ST2 (35.8 ± 10.7) and the SHM groups (32.0 ± 8.9 pg/mg protein) (*p* < 0.001; Figure 2(c)). Also, in the EDL muscle, the concentration of 8-iso was elevated approximately fourfold in the ST4 group as compared to the SHM and ST2 groups (*p* < 0.001Figure 2(d)).

#### 3.2.2. Marker of Protein Oxidation

**Figure 3:**
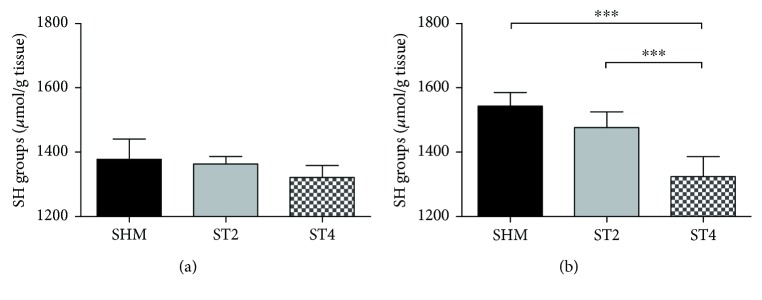
The level of SH groups in (a) SOL and (b) EDL. Results were expressed as mean ± SD, SHM (*n* = 6), ST2 (*n* = 6), ST4 (*n* = 5). ^∗∗∗^*p* < 0.001.

The level of SH group was not significantly different between groups in SOL muscle after the BST stimulation (Figure 3) and it was 1321.2 in the ST4, 1363.1 in the ST2, and 1378.2 *μ*mol/g tissue in the SHM group, respectively. However, in the EDL muscle, the level of SH groups in the ST4 rats was significantly lower than in the SHM group, also differences in the ST4 as compared to the ST2 group was observed in the EDL muscle after the electrical stimulation (*p* < 0.001; Figure 3(b)). The values of SH groups were 1323.5 ± 62.1, 1476.9 ± 48.7, and 1543.3 ± 41.8 *μ*mol/g tissue in the ST4, ST2, and SHM groups, respectively.

**Figure 4:**
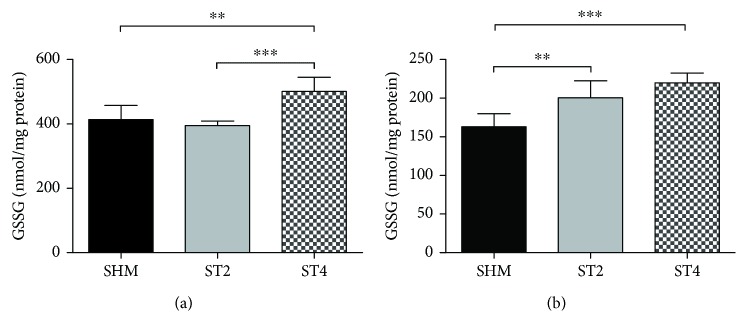
The level of GSSG in (a) SOL and (b) EDL. Results were expressed as mean ± SD, SHM (*n* = 6), ST2 (*n* = 6), ST4 (*n* = 5). ^∗∗^*p* < 0.01, ^∗∗∗^*p* < 0.001.

The concentration of GSSG was the highest in the ST4 group in both muscles. In the SOL muscles, there were 500.9 ± 43.8 in the ST4, 395.4 ± 13.8 in the ST2, and 413.9 ± 43.5 nmol/mg protein in the SHM groups, respectively (*p* < 0.001 ST4 versus ST2; *p* < 0.01 ST4 versus SHM) (Figure 4(a)). There was significantly higher level of GSSG in both the ST4 and ST2 in the EDL muscle, as compared to the SHM group (*p* < 0.001 ST4 versus SHM *p* < 0.01 ST2 versus SHM). The values were 219.8 ± 12.9, 200.6 ± 22.1, and 163.0 ± 16.9 nmol/mg protein in the ST4, ST2, and SHM groups, respectively (Figure 4(b)).

#### 3.2.3. Antioxidant Enzyme Activity

**Figure 5:**
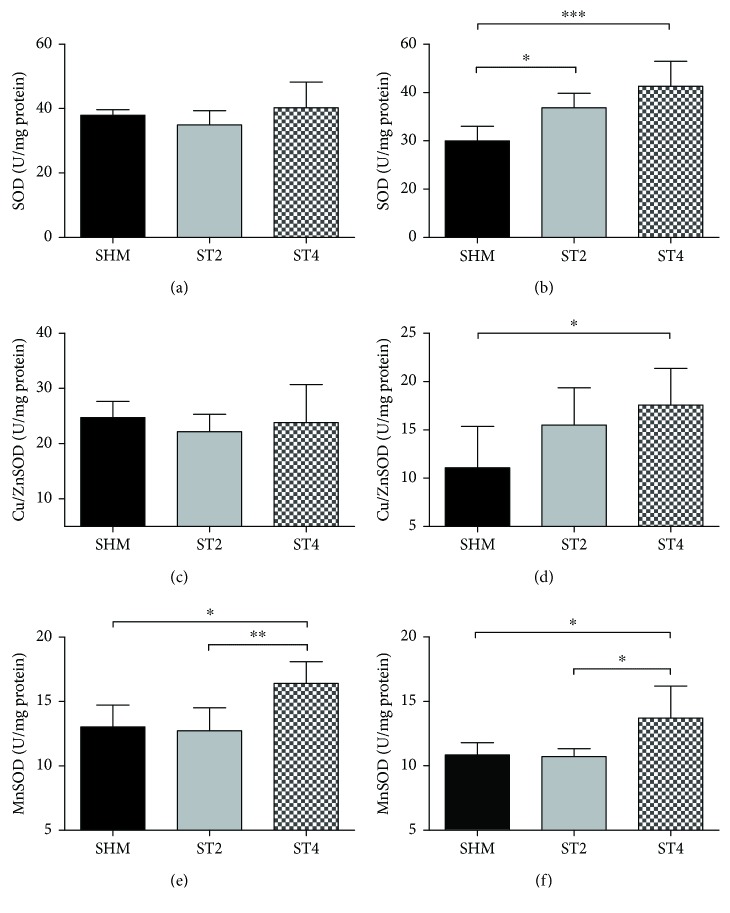
The activity of total SOD in (a) SOL and (b) EDL, Cu/ZnSOD in (c) SOL and (d) EDL, and MnSOD in (e) SOL and (f) EDL. Results were expressed as mean ± SD, SHM (*n* = 6), ST2 (*n* = 6), ST4 (*n* = 5). ^∗^*p* < 0.05, ^∗∗^*p* < 0.01, ^∗∗∗^*p* < 0.001.

The activity of total SOD was higher in the ST4 group compared to the SHM group only in the EDL muscle (*p* < 0.001; Figure 5(b)). Total SOD activity was also elevated in the ST2 group versus the SHM group in the EDL muscle (*p* < 0.05; Figure 5(b)). The activity of total SOD was not different in the SOL muscle after rat stimulation (Figure 5(a)). In Cu/Zn SOD activity, there were no differences between groups in SOL muscle (Figure 5). However, the EDL muscle activity of Cu/ZnSOD was higher in the ST4 as compared to the SHM group (*p* < 0.05; Figure 5(d)). The mitochondrial isoform of SOD activity was higher in the ST4 group than in both ST2 and SHM groups in the SOL muscle (*p* < 0.01 and *p* < 0.05, resp.; Figure 5(e)). There was also elevated MnSOD activity in the ST4 group versus both the ST2 and SHM groups in EDL muscle (*p* < 0.05; Figure 5(f)).

**Figure 6:**
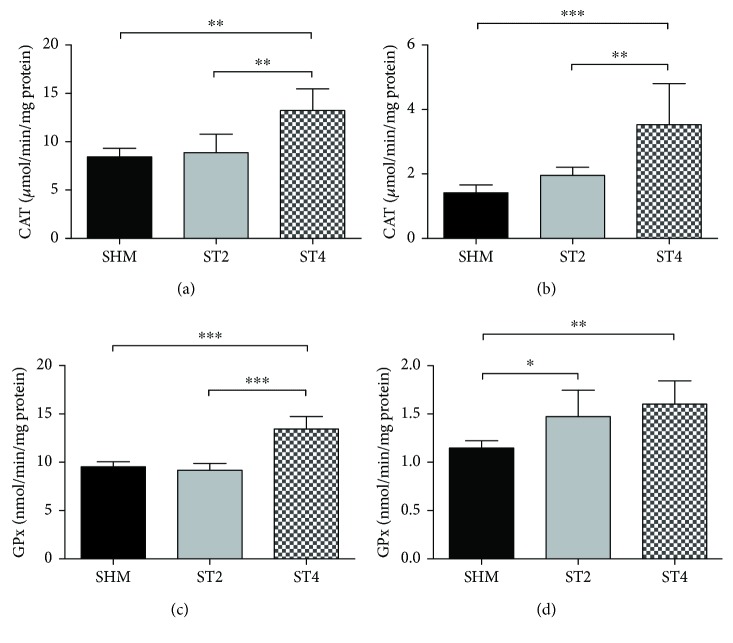
The activity of CAT in (a) SOL and (b) EDL. The activity of GPx in (c) SOL and (d) EDL. Results were expressed as mean ± SD, SHM (*n* = 6), ST2 (*n* = 6), ST4 (*n* = 5). ^∗^*p* < 0.05, ^∗∗^*p* < 0.01, ^∗∗∗^*p* < 0.001.

After 4 weeks of rat stimulation, CAT activity was higher in the ST4 group when compared to the ST2 and SHM groups in both muscles. In the SOL muscle, the activity of CAT was 8.4 ± 0.9 in the SHM group, 8.9 ± 1.9 in the ST2 group, and 13.2 ± 2.3 *μ*mol/min/mg of protein in the ST4 group (*p* < 0.01; ST4 versus ST2 and ST4 versus SHM) (Figure 6(a)). The activity of CAT in the EDL muscle was lower than in the SOL muscle; nevertheless, differences between groups were also significant (*p* < 0.01 ST4 versus ST2, *p* < 0.001 ST4 versus SHM). The CAT activity was 1.4 ± 0.2 in the SHM group, 2.0 ± 0.3 in the ST2 group, and 3.5 ± 1.3 *μ*mol/min/mg of protein in the ST4 group in the EDL muscle after the electrical stimulation of the BST (Figure 6(b)).

GPx activity was significantly higher in the ST4 group compared to the SHM group, both in the SOL and EDL muscles. In the SOL muscle, the value was 13.4 ± 1.3 in the ST4 group, 9.2 ± 0.7 in the ST2 group, and 9.5 ± 0.5 nmol/min/mg protein in the SHM group (*p* < 0.001 ST4 versus ST2 and ST4 versus SHM; Figure 6(c)). In the EDL muscle, significant differences were observed between the ST4 and the SHM groups (*p* < 0.01; Figure 6(d)) and between the ST2 and the SHM groups (*p* < 0.05). The average activity of GPx was 1.6 ± 0.2, 1.5 ± 0.3, and 1.1 ± 0.1 nmol/min/mg of protein in the ST4, ST2, and SHM groups, respectively.

## 4. Discussion

To the best of our knowledge, this is the first preclinical study showing that the 4-week electrical stimulation of the BST considerably induces HPA axis activation and evokes CORT secretion. In addition, the elevated plasma CORT concentration was associated with increased oxidative stress in skeletal muscle. We found higher concentration of markers of lipid and protein peroxidation and elevated activity of antioxidant enzymes in both SOL and EDL muscles after 4 weeks of stimulation. Moreover, we did not observe similar changes after 2 weeks of stimulation, short stress response (SSR). This observation is consistent with earlier data conducted by Myślińska and coworkers, where a lack of differences was observed in plasma CORT level between the sham-operated and 2-week BST-stimulated groups [25]. Taking into account anatomical and functional basis of the inductive role of the BST in HPA axis activation [19, 20] with simultaneous no differences in plasma CORT level after 2 weeks of the BST stimulation in experimental condition, we decided to extend the time of the electrical stimulation of this limbic structure. Our data suggest that exposure to the CSR (4 weeks of stimulation) is associated with free radical damage of macromolecules in skeletal muscle. In the present study, we noticed fivefold increase of the CORT level in the ST4 group versus the SHM group. It may prove the essential role of the BST in the modulation of CSR, what was widely discussed before [19, 21]. Moreover, obtained data suggest that the chronic 4-week electrical stimulation of BST could mimic long-term mental stress and be useful as a “model” of unconscious stress. In the study by Fontella and coworkers, it was demonstrated that repeated restraint stress entails the increase of plasma CORT level [13]. What is more, another study showed two times higher (compared with [13] and the current study) release of the CORT after restraint stress [12]. Despite the differences in particular data, the multifold increase of plasma CORT level compared with the control group seems to confirm credibility of adopted model of stress response.

### 4.1. The Exogenous GC Administration as a Mimic of Stress Response

One of the considered methods used to mimic the stress response is an exogenous CORT or other synthetic GC administration. Oshima and coworkers showed that treatment with dexamethasone (DEX) leads to an increased generation of ROS in both human rhabdomyosarcoma and a dopaminergic neuroblastoma cell lines [6]. Also, it was reported that DEX treatment rats decreased GSH content in blood and SOL muscle [16] and GSH/GSSG ratio decreased in the brain in TDP-25 transgenic mice [31]. In the current study, similar effect was observed, where the level of GSSG in both types of muscle was the highest in the ST4 group. Likewise, it was reported that lipid peroxidation markers, TBARs or MDA, were increased in blood [16], brain [32, 33], lymphoid organs [34], and even seminal plasma [35] after DEX administration. In the experiment conducted by Pereira and coworkers, higher concentration of TBARs was presented in both gastrocnemius and SOL muscles after just three days of DEX injections versus control group [34]. On the other hand, Jeje and Raji reported higher liver MDA level after chronic 14 and 21 days of DEX treatment when compared with short-term DEX administration [14]. Additionally, it was shown that oxidative stress greatly increased with duration of GC administration, mostly after 3 weeks of treatment [36]. Therefore, in the current study, we expected that CSR would have greater impact on oxidative stress marker level than SSR. We found higher concentration of MDA and 8-iso in both SOL and EDL muscles. Furthermore, our data also showed that marker of protein oxidation, SH groups, was reduced only in the EDL muscle. This observation is consistent with earlier reports documented that white muscles are more susceptible to free radical damage [30]. Moreover, the susceptibility for oxidation of white muscle might be displayed with the result of GSSG level, where the elevation was observed not only in the ST4 but also in the ST2 group. Our data are in line with earlier observations that ROS generation was higher in white muscle versus red muscle [30, 37]. Additionally, it was demonstrated that the EDL muscle shows a greater tendency to atrophy after DEX treatment than the SOL muscle [38]. One of the explanations of this phenomenon can be that the fast-twitch muscles contain higher glucocorticoid receptor (GR) content compared to slow-twitch muscles [39], and therefore it is presumably more susceptible to develop negative effect of GC action. However, opposite conclusion was demonstrated [40], it was suggested that an increased sensitivity of the SOL muscle to GCs occurs only in old rats.

### 4.2. The Endogenous GC Secretion as a Base of Stress Response

Recently, it was shown that GC secretion might be provoked by different types of stressors [41]. Some of them link oxidative stress in skeletal muscles with hypermetabolism state and with stress response to situations such as burn trauma [42, 43]. However, other pathological conditions, for example, cancer, may stimulate endogenous GC production and contribute to metabolic derangements and the skeletal muscle loss [44, 45]. Likewise, psychological stress is widely associated with ROS generation, mainly in central nervous system, with simultaneous increase of plasma CORT level. Elevated GCs is combined with the development of impairments of cognitive function, learning, and memory [10–13, 33, 46–48]. The evidence for a direct connection was observed between CSR and neurodegenerative disorders such as AD or PD [49–51]. Moreover, one of the earliest events in AD pathogenesis is a systemic oxidative stress, indicated by an increase lipid peroxidation and GSSG in cerebrospinal fluid, plasma, and urine [52]. Taken together, it seems to be worth to consider that both AD and PD show abnormalities not only in structure of central nervous system but also in abnormal functioning of skeletal muscle which cause disorders such as bradykinesia, rigidity, tremor, postural instability, and gait disorders [53, 54]. Therefore, wider perspective to the assessment of oxidative stress in skeletal muscle in neurodegenerative diseases seems to be justified.

### 4.3. The Antioxidant Enzyme Activity Changes during Stress Response

**Figure 7:**
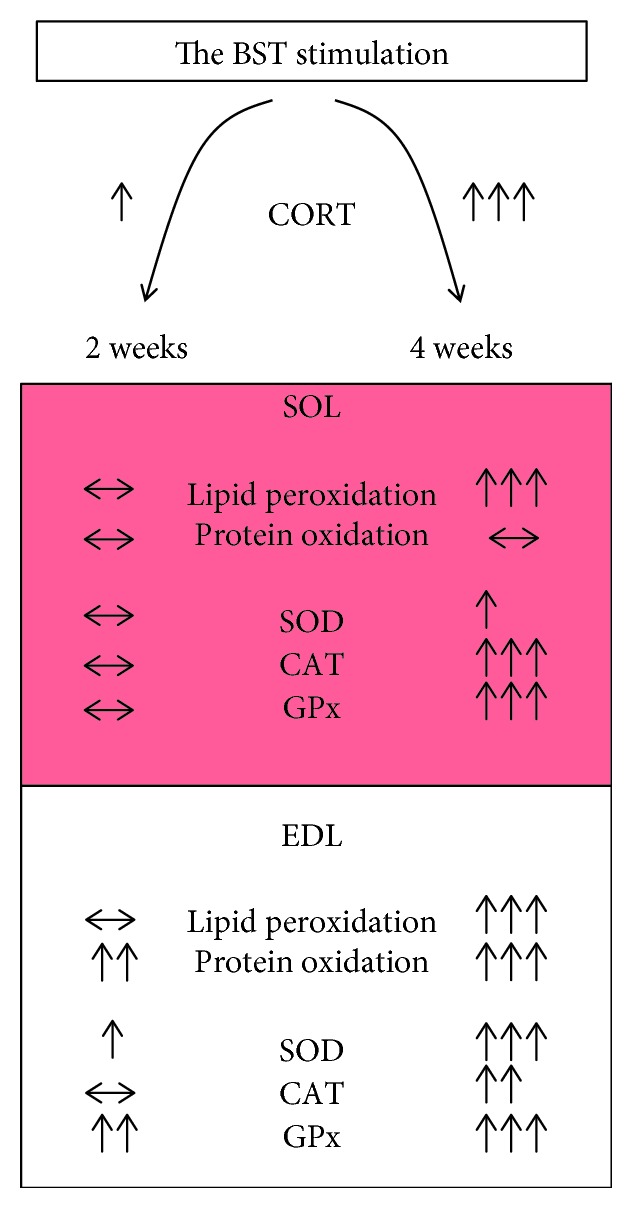
The electrical stimulation of BST activates HPA axis and induces oxidative stress in both types of skeletal muscles during CSR.

In the current study, we observed increased activity of main three antioxidant enzymes in the white muscle and two of them (CAT and GPx) in the red muscle. We did not observe any changes in the activity of total and Cu/ZnSOD in the SOL muscle. In contrast with the red muscle, EDL activity of total SOD and both Cu/ZnSOD and MnSOD significantly increased as compared to the SHM and ST2 groups after 4 weeks of stimulation. Further, in the red muscle, GPx activity was higher only in 4-week stimulated group. Interesting observations were made in the white muscle where the activity of GPx was higher not only in the ST4 group but also in the ST2 group compared to the SHM group. This observation corresponds with the level of GSSG and confirms that the EDL muscle is more susceptible to the disruption than the SOL muscle. However, an opposite effect was observed in the studies on the influence of CORT injections on ROS generation in the hippocampus where activity of SOD, CAT, and GPx decreased after CORT treatment compared to a control group [10]. One of the explanations for these differences in antioxidant enzyme activities between the brain and muscle can be that the brain is much more susceptible to GC-induced oxidative stress. Also, the brain is the main target of GCs and generally has low antioxidant capacity, high metabolic activity, and highly susceptible to peroxidation cell membranes [36]. Secondly, it is also well known that differences between red and white muscles occur, and red muscles possess higher antioxidative capacity in terms of higher antioxidant enzyme activity [55], what may provide more effective defense against ROS generation. Another explanation for the increased activity of antioxidant enzymes in our study may be a pathway where ROS induced by GCs activate a signaling cascade, which results in further FOXO activation [9]. It was shown that FOXO may induce gene expression for antioxidant enzymes, that is, MnSOD and CAT but also proteins responsible for protein catabolism [3, 52]. To our knowledge, this is the first study that shows the influence of BST on regulation of HPA axis activity during CSR with simultaneous oxidative stress in both types of skeletal muscles is a consequence of elevated plasma CORT level (Figure 7).

## 5. Conclusions

**Figure 8:**
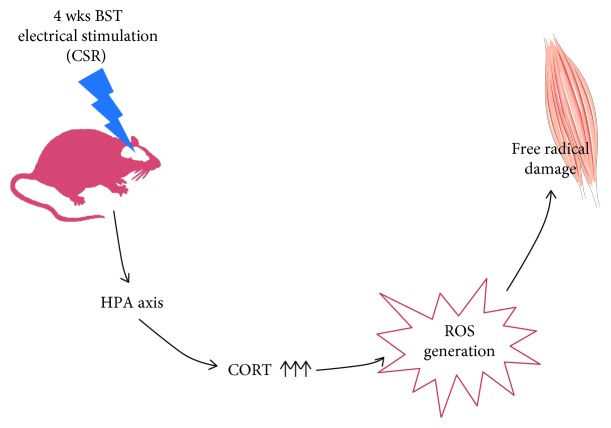
The potential consequences of reaction to the long-term “psychological stress” lead to the free radical damage of skeletal muscle. (1) The bed nucleus of the stria terminalis is linked with the chronic stress response. (2) The chronic BST stimulation induces massive plasma CORT secretion by HPA axis. (3) The chronic stress response evokes ROS generation in two types of skeletal muscles. (4) Lipid and protein peroxidation in muscle is associated with higher CORT level. (5) The activity of antioxidant enzymes is elevated during the chronic stress response.

We found elevated level of lipid and protein peroxidation and higher activity of antioxidant enzymes in both SOL and EDL muscles after 4 weeks of stimulation. Moreover, four but not two weeks of electrical stimulation of BST evokes increased plasma CORT level, resulted in oxidative stress in skeletal muscles (Figure 8). Taken into account, higher markers of free radical damage of macromolecules and elevated antioxidant enzyme activity in EDL muscle, we determine that the white muscle is more susceptible for disruption. These data may suggest that the electrical stimulation of the BST could be useful as a novel imitator of the CSR. Therefore, we postulate that the CSR associated with ROS generation may be one of the factors for the development of neurodegenerative diseases in human being. However, further studies are necessary to find the mechanism(s) of macromolecule disruption resulted by the CSR.

## Data Availability

The data used to support the findings of this study are available from the corresponding author upon request.

## Abbreviations

BST: Bed nucleus of the stria terminalis
CAT: Catalase
CORT: Corticosterone
Cu/ZnSOD: Copper/zinc-dependent dismutase
CSR: Chronic stress response
DEX: Dexamethasone
GCs: Glucocorticoids
GPx: Glutathione peroxidase
GR: Glucocorticoid receptor
GSSG: Glutathione disulfide
MDA: Malondialdehyde
MnSOD: Manganese-dependent superoxide dismutase
ROS: Reactive oxygen species
SH: Sulfhydryl groups
SSR: Short stress response.
